# Identification of a novel gene fusion (*BMX-ARHGAP*) in gastric cardia adenocarcinoma

**DOI:** 10.1186/s13000-014-0218-4

**Published:** 2014-12-13

**Authors:** Xiaofeng Xu, Lifang Xu, Feng Gao, Jianjiang Wang, Jinsong Ye, Mingxian Zhou, Yunling Zhu, Lan Tao

**Affiliations:** Clinical Laboratory, People’s hospital, Jingjiang, 214500 Jiangsu China; Department of Gastroenterology, People’s hospital, Jingjiang, 214500 Jiangsu China; Department of General Surgery, People’s hospital, Jingjiang, 214500 Jiangsu China

**Keywords:** Gastric cardia adenocarcinoma (GCA), RNA-Seq, Gene fusion

## Abstract

**Background:**

Gastric cardia adenocarcinoma (GCA) is one of the major causes of cancer related mortality worldwide. We aim to provide new understanding in the pathogenesis of GCA through investigations on gene expression alterations.

**Methods:**

We preformed RNA-Seq for one pair of GCA and matched non-tumor tissues. Differentially expressed genes (DEGs) and fusion genes were acquired. PCR and gel analysis in additional 14 pairs of samples were performed to validate the chimeric transcripts.

**Results:**

1590 up-regulated and 709 down-regulated genes were detected. Functional analysis revealed that these DEGs were significantly overrepresented in gene ontology items of cell cycle, tumor invasion and proliferation. Moreover, we firstly discovered 3 fusion genes in GCA, including *BMX-ARHGAP*, *LRP5- LITAF* and *CBX3-C15orf57*. The chimeric transcript *BMX-ARHGAP* was validated and recurrently occurred in 4/15 independent tumor tissues.

**Conclusions:**

Our results may provide new understanding of GCA and biomarkers for further therapeutic studies.

**Virtual Slides:**

The virtual slide(s) for this article can be found here: http://www.diagnosticpathology.diagnomx.eu/vs/13000_2014_218

## Background

Gastric cancer is the fourth most common malignant cancer and the second major cause of cancer-related death [1,2]. It is widely believed that gastric cancer is a heterogeneous disease with multiple environmental and genetic etiologies. To date, aberrant gene expression and epigenetic alterations were identified to be involved in the pathogenesis of gastric cancer. These abnormal changes could lead to perturbations in normal cellular homeostasis and neoplastic transformation of the gastric mucosa [3]. In particular, disruption in a number of regulatory signal pathways could create a permissive environment for carcinogenesis, invasiveness and metastasis. Gastric adenocarcinoma comprises 95% of the malignant gastric tumors [4]. It is classified as proximal (originating in the cardia) and distal (originating distal to the cardia). Different from distal adenocarcinomas, incidence of gastric cardia adenocarcinoma (GCA) has increased significantly recently [5-7]. In addition, compared with distal adenocarcinomas, GCA seems to be more aggressive with deeper gastric wall invasion and worse prognosis [5,8]. Therefore, it is necessary to spend more efforts on the investigations for uncovering the pathogenesis of GCA.

With the rapid development of next generation sequencing (NGS), many cancer-related genes have been identified. NGS technology makes it possible to comprehensively illuminate whole map of genetic alteration of cancer. Specifically, massively parallel RNA-Sequencing (RNA-Seq) allows identification of entire gene expression and structural variation in individual samples, and facilitates fully characterization of cellular transcriptomes. Consequently, RNA-Seq becomes a revolutionary tool to study transcriptome profiling and measure the expression levels of various transcripts and isoforms [9]. Currently, investigations on GCA by using RNA-seq are still limited.

In this study, we generated comprehensive mRNA profiles in a pair of GCA and adjacent non-tumor tissues. We performed transcriptome-wide, unbiased analyses of the RNA-Seq data to identify different kinds of gene transcriptional aberrations (mRNA expression and chimeric transcript) to investigate the molecular mechanism of gastric cancer pathogenesis. Interesting fusion genes were further validated in other independent samples. Our results may provide new understanding of GCA pathogenesis and new targets for future therapeutic studies.

## Methods

### Sample information

The GCA tissue and adjacent non-tumor tissue (5 cm away from tumor) were collected from a 65 years old male patient who was diagnosed as T4N0M0 stage IIB GCA in 2012. The tumor size was 5 × 4 × 1 cm. The tumor was moderately differentiated and didn’t spread to nearby lymph nodes or other organs. However, both venous invasion and nerve invasion were positive.

For validation of interesting fusion genes, another 14 pairs of stage IIB GCA and adjacent non-tumor tissues were obtained with the same procedure. Signed informed consent documents were obtained from all patients. The scientific use of these samples was approved by the Institutional Review Boards of the People’s hospital, Jingjiang, Jiangsu, China.

### RNA-Seq library preparation and Illumina sequencing

Total RNA was isolated from frozen tissue by Trizol (Invotrogen) and its quality was assessed using Agilent Bioanalyzer. RNA-Seq libraries were prepared by TruSeq RNA Sample Prep Kit (Illumina Cat. No. FC-122-1001) according to standard protocols. Then the Libraries were sequenced on an Illumina Genome Analyzer IIx with 115 bp pair-end read length.

### RNA-Seq data processing

For the raw data, low quality reads were filtered according to the criteria as follows: (1) reads containing adaptors were filtered; (2) nucleotides with a quality score lower than 20 were trimmed. The clean reads were aligned against both genome hg19 and transcripts reference using Bowtie 2.0.0 [10] and Tophat1.3.1 [11]. The mapping reads without biological interest, such as ribosomal RNAs and mitochondrial RNA were removed. The results of read mapping was visualized by using Integrative Genomics Viewer (IGV 2.0.26) [12].

### Detection of differentially expressed genes (DEGs)

To identify DEGs, the values of human genes were normalized by per kilobase of exon per million mapped reads (FPKM) using Cufflinks (1.0.3) [13]. DEGs between GCA and non-tumor tissues were determined with significance cutoff of q-value <0.05. In addition, gene ontology (GO) and KEGG pathway enrichment analyses were performed via the web tool DAVID (http://david.abcc.ncifcrf.gov/) [14] with the false discovery rate (FDR) <0.05.

### Alternative splicing event detection

The alternative splicing events were detected by MISO software [15], including alternative 3’ splice sites (A3SS), alternative 5’ splice sites (A5SS), mutually exclusive exons (MXE), retained intron (RI) and skipped exons (SE). Differentially expressed isoforms were further identified with following criteria: 1) the value of gene expression difference >0.2; 2) Bayes factors >10; 3) inclusive reads >1, exclusive reads > 1 and sum of inclusive reads and exclusive reads >10.

### Fusion gene identification

Fusion gene was identified by Defuse [16] and TopHat [17]. The filtering processes of Defuse were carried out as previously described [18]. Fusion genes detected by TopHat should meet the following criteria: 1) support reads number should be more than 3; 2) the supporting reads should not be mapped to ribosomal protein or small nuclear ribonucleoproteins. Only fusion genes detected by both methods were remained.

### Gene fusion validation

To validate fusion transcripts, we performed PCR and gel analysis in the sequence sample and another 14 pairs of GCA and adjacent non-tumor tissues. All patients were also diagnosed as T4N0M0 stage IIB GCA. Primer pairs were designed using Primer 5 software, and RT-PCR was performed using the following procedure: 94°C for 1 min, 40 cycles of 94°C for 20 sec, 55°C for 20 sec and 72°C for 15 sec, followed by 72°C for 1 min. The PCR products of the fusion genes were visualized through agarose gel electrophoresis.

## Results

**Figure 1:**
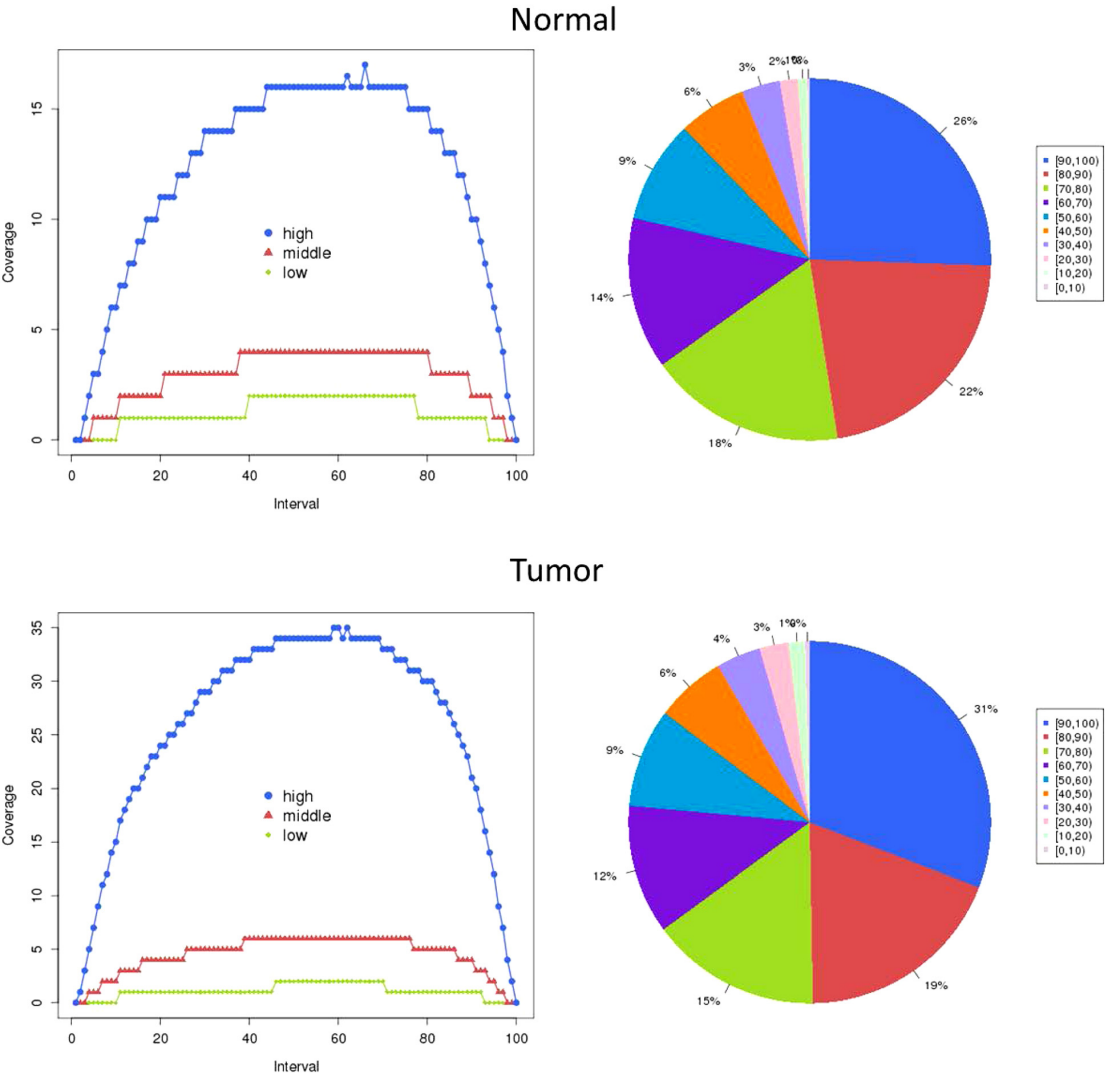
**The evenness (left) and integrity (right) of reads distribution.** The left two figures show the reads distribution in different parts of genes. The X-axis represents that the gene body is divided into 100 parts from 5’ end to 3’ end. The Y-axis represents the number of reads in different parts. The right two figures show the reads coverage rate distribution in genes. Coverage rate is count by reads covered gene length divided by total length of the gene.

In order to explore the spectrum of gene transcriptional changes in GCA, we sequenced the whole gene transcripts in one pair of GCA and matched non-tumor tissues from a 65 years old patient. We generated a total of 5.36 and 6.12 Gb data for the tumor and non-tumor tissue, respectively. Approximately 80% reads were mapped to the reference genome, achieving an average depth of coverage of 38.63 X and 43.32 X, respectively (Table 1). The sequencing reads achieved fairly well evenness and integrity (Figure 1).

**Table 1:** **Statistics of the sequenced reads and its mapping status**

**Sample**	**Total reads**	**Mapped reads**	**Mapping ratio**	**Total base(bp)**	**Mapped base(bp)**	**Coverage(×)**
Non-tumor	53611554	44218606	82.48%	5361155400	4421860600	38.63
Tumor	61217124	49477807	80.82%	6121712400	4947780700	43.22

Next we analyzed gene expression level via calculating FPKM using the Cufflinks. About 15 thousand genes were expressed (FPKM >1) in our samples, among which 1590 genes were up-regulated and 709 genes were down-regulated in tumor. According to the enrichment analysis, these DEGs were significant overrepresented in 16 GO terms and 3 pathways, which were related to cell cycle, tumor invasion and proliferation (Table 2).

**Table 2:** **Gene ontology items and pathways that significantly overrepresented with differentially expressed genes**

**ID**	**Description**	***P*** **value**
**Gene ontology**		
GO:0022403	cell cycle phase	2.11E-15
GO:0000279	M phase	4.11E-15
GO:0007049	cell cycle	7.68E-14
GO:0000280	nuclear division	1.87E-13
GO:0007067	mitosis	1.87E-13
GO:0048285	organelle fission	4.94E-13
GO:0022402	cell cycle process	5.06E-13
GO:0000087	M phase of mitotic cell cycle	5.06E-13
GO:0000278	mitotic cell cycle	6.84E-11
GO:0051301	cell division	4.74E-10
GO:0042127	regulation of cell proliferation	6.42E-07
GO:0007155	cell adhesion	1.47E-06
GO:0022610	biological adhesion	1.59E-06
GO:0007059	chromosome segregation	1.47E-05
GO:0007051	spindle organization	1.99E-05
GO:0007010	cytoskeleton organization	3.29E-05
**KEGG pathway**		
hsa05130	Pathogenic Escherichia coli infection	9.84E-03
hsa04512	ECM-receptor interaction	1.29E-02
hsa04110	Cell cycle	1.86E-02

Alternative splicing events were also detected by using MISO. We found 311 differentially alternative spliced events. After the filtering process, 61 alternative splicing events were remained according to Bayes factors, Psi values and confidence intervals (Table 3).

**Table 3:** **The differentially alternative splicing events**

	**A3SS**	**A5SS**	**MXE**	**RI**	**SE**	**Total**
Tumor vs. Non-tumor (raw)	31	38	53	75	114	311
Tumor vs. Non-tumor (filtered)	5	10	11	12	23	61

**Figure 2:**
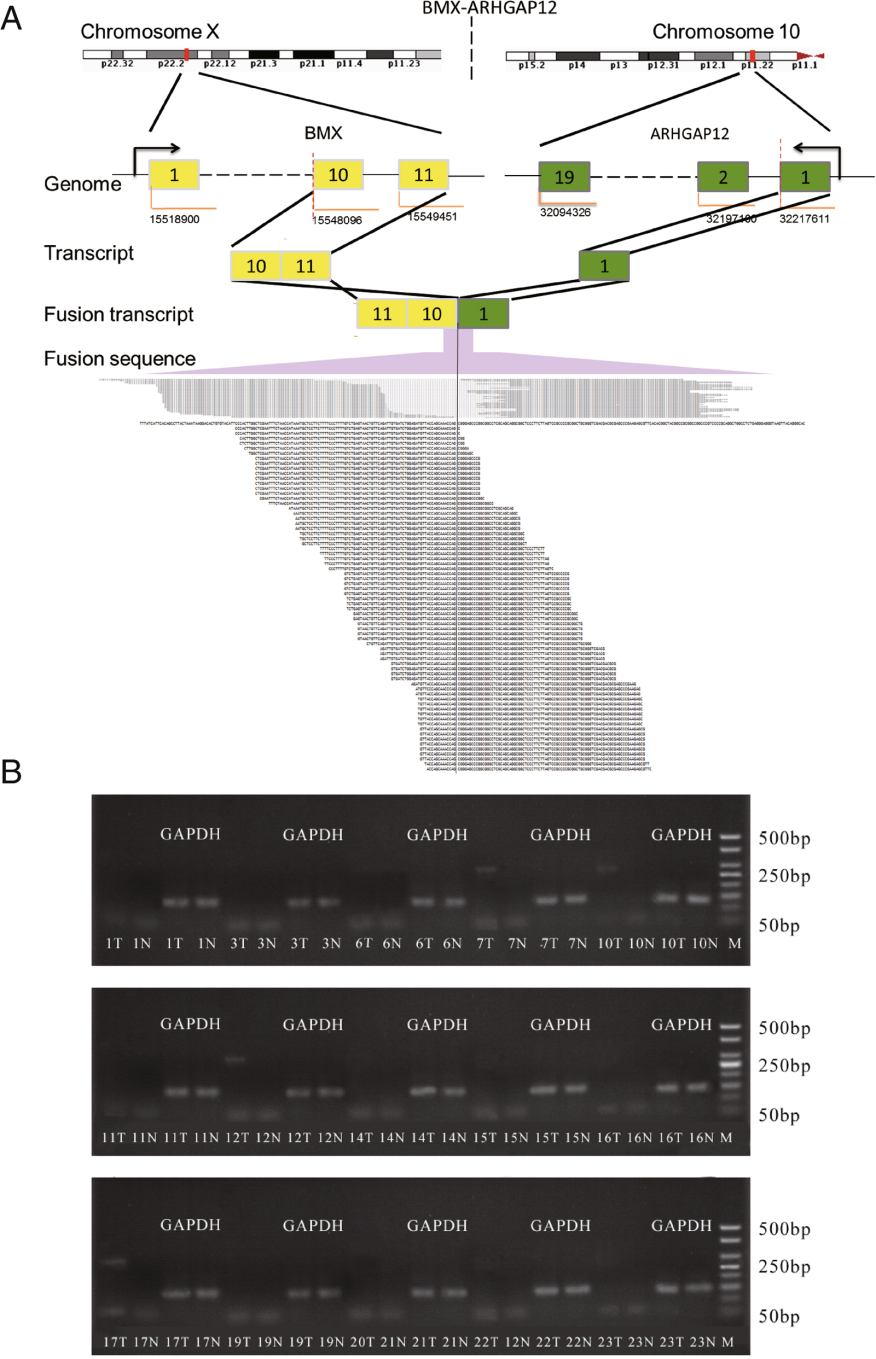
**Detection and validation of the fusion gene** ***BMX-ARHGAP*** **in gastric cardia adenocarcinoma. A**. *BMX* locates in chromosome X and *ARHGAP* locates in chromosome 10. The breakpoint of *BMX-ARHGAP* is supported by both paired reads and single reads. **B**. Validation of the gene fusion with the target band of 260 bp. Sample 10 is the sequencing sample. T and N represent tumor and non-tumor tissue, respectively. It is obviously the expected target band is present in the tumor tissues of sample 7, 10, 12, and 17.

Many fusion genes were reported as the potential cause for tumorigenesis, including gastric cancer [19,20]. Using Defuse and TopHat, 7 candidate fusion genes were identified with stringent filtering criteria. And finally 3 fusion genes were selected by TopHat realignment and manual inspection (Table 4, Figure 2). The fusion gene *BMX-ARHGAP* was validated using RT-PCR and gel analysis (Table 5), which was recurrently present in tumor tissues in about 26.7% (4/15) GCA patients (Figure 2).

**Table 4:** **Summary of candidate fusion genes detected by RNA-Seq**

**No.**	**Gene1**	**Gene2**	**Break point1**	**Break point2**
1	*BMX*	*ARHGAP12*	chrX:15548096	chr10:32217611
2	*LRP5*	*LITAF*	chr11:68125315	chr16:11650591
3	*C15orf57*	*CBX3*	chr15:40854974	chr7:26241386

**Table 5:** **The primer sequences used in PCR and gene fusion validation**

**Gene**	**Forward (5’-3’)**	**Reverse (5’-3’)**
GAPDH*	CATGAGAAGTATGACAACAGCCT	AGTCCTTCCACGATACCAAAGT
^$^ *BMX*	GTGTACATTCCCACTTGGCTCG	
^$^ *ARHGAP12*		CCTGTAACTTACCCTCCCTCAG

*the control; ^$^primer used to amplify gene fusion.

## Discussion

The development of cancer is a multistep process during which cells acquires a series of mutations that eventually lead to unrestrained cell growth and division, inhibition of cell differentiation, and evasion of cell death. In order to comprehensively study aberrant gene expression in GCA, we performed the current study by using paired-end RNA-Seq technology. The results revealed the information of DEGs, alternative splicing, and gene fusion, which may have potential application in therapeutic studies.

In the present study, over 400 M reads were sequenced on the Illumina platform, reaching about 40 X coverage for the whole transcriptome. With those high sequencing depth, 2299 DEGs were detected between GCA and non-tumor tissue. Further analysis showed ECM and cell cycle were the most enriched biological pathway among those abnormal expressed genes.

Alternative splicing is an important regulatory process during gene expression and substantially results in diverse transcripts. Abnormally spliced mRNAs were also found in multiple cancerous cells, including gastric cancer, breast cancer, and colon cancer [21,22]. In our study, 61 alternative spliced events were identified by using MISO software. Among them, *CD44* was also found be abnormally spliced in colorectal cancer and was suggested to be the character of metastatically potent tumor cells [23].

Gene fusion is another oncogenic activation mechanisms involved in the development of various types of malignancies including leukemia, lymphoma, breast and prostate cancer [24]. Importantly, several fusion genes, *DUS4L–BCAP29* [19], *CD44-SLC1A2* [20] have been reported in gastric cancer in western country. In our study, 3 chimeric transcripts were supported by the two detection methods (Table 4). Among them, *CBX3-C15orf57* was detected in healthy human beings before [25]. In addition, both *CBX3* and *C15orf57* were recurrently partnered with others in breast cancer [26]. Our results implicate the potential involvement of *CBX3-C15orf57* in GCA. *BMX-ARHGAP12* was firstly validated and was detected recurrently in 4 out of 15 GCA patients (Figure 2). *BMX* encodes a non-receptor tyrosine kinase and *ARHGAP12* encodes a Rho GTPase activating protein. It is possible that the chimeric transcript active the BMX mediation of tumorigenicity in GCA patients since BMX has been suggested to promote tumor growth and metastasis other cancers [27,28]. In addition, overexpression of ARHGAP12 would impair cell scattering, invasion and adhesion to fibronectin [29]. This fusion gene may play important roles in the tumorigenesis and may be potential targets for novel therapeutic strategy.

## Conclusions

In summary, we performed transcriptome-wide analysis to identify gene expression aberrations in GCA. One of identified fusion gene, *BMX-ARHGAP12*, was further confirmed in another independent patient. Our results may provide new understanding of the pathogenesis and new targets for further therapeutic investigations.
